# The Potential of MSC-Based Cell-Free Therapy in Wound Healing—A Thorough Literature Review

**DOI:** 10.3390/ijms24119356

**Published:** 2023-05-27

**Authors:** Hui Ma, Wing-Sum Siu, Ping-Chung Leung

**Affiliations:** 1 Institute of Chinese Medicine, The Chinese University of Hong Kong, Shatin, New Territories, Hong Kong SAR, China; huima@cuhk.edu.hk (H.M.); sammysiu@cuhk.edu.hk (W.-S.S.); 2 State Key Laboratory of Research on Bioactivities and Clinical Applications of Medicinal Plants, The Chinese University of Hong Kong, Shatin, New Territories, Hong Kong SAR, China

**Keywords:** mesenchymal stem cells (MSCs), cell-free, wound healing, secretome, exosome

## Abstract

A wound is an interruption of the normal anatomic structure and function of the skin, which is critical in protecting against foreign pathogens, regulating body temperature and water balance. Wound healing is a complex process involving various phases, including coagulation, inflammation, angiogenesis, re-epithelialization, and re-modeling. Factors such as infection, ischemia, and chronic diseases such as diabetes can compromise wound healing, leading to chronic and refractory ulcers. Mesenchymal stem cells (MSCs) have been used to treat various wound models due to their paracrine activity (secretome) and extracellular vehicles (exosomes) that contain several molecules, including long non-coding RNAs (lncRNAs), micro-RNAs (miRNAs), proteins, and lipids. Studies have shown that MSCs-based cell-free therapy using secretome and exosomes has great potential in regenerative medicine compared to MSCs, as there are fewer safety concerns. This review provides an overview of the pathophysiology of cutaneous wounds and the potential of MSCs-based cell-free therapy in each phase of wound healing. It also discusses clinical studies of MSCs-based cell-free therapies.

## 1. Introduction

A skin wound is caused by the disruption of the epidermal layer integrity [1]. Wound healing is activated right after the skin damage, but it is not a rapid process. It consists of a complicated procedure, including a series of well-organized cellular and molecular events [2]. Any disruption in the cascade of events causes failure in wound healing [3].

Wound healing can be classified as primary healing and secondary healing. Primary healing is an uncomplicated healing [4]. It occurs in aseptic wounds with smooth borders, surgical wounds for instance. Generally, it heals within 6–8 days. Secondary healing starts when the wound is disrupted with large tissue losses or complicated with infection. Granulation tissue forms, leading to scarring. It takes longer time with higher risk of infections and poor healing [5].

### 1.1. Acute and Chronic Wounds

Based on the pathogenesis and consequences, skin wounds can be classified as acute and chronic wounds [6]. An acute wound progresses through the process of a normal wound healing, resulting in wound closure without disruption. Commonly, it follows surgical incision or trauma, such as thermal wounds, abrasions, and lacerations. It heals in a timely and orderly manner [7]. The healing of acute wound is regulated by cytokines and different growth factors. Neutrophil, macrophage, and lymphocyte migration are involved in the inflammatory phase, which lasts for days to weeks [8]. Chronic wound is defined when a wound fails to heal in 4 weeks [9]. Several factors and disease conditions, such as age, hormones, immune status, psychosocial issues, genetic conditions, malnutrition, infection, insufficient oxygenation or perfusion, smoking, medications, radiation, and chemotherapy, could impair the healing process and lead to persistent pro-inflammatory condition [10]. The chronic wounds usually include vascular ulcers (venous or arterial ulcers), diabetic ulcers, pressure ulcers, and other refractory ulcers [11].

### 1.2. Different Phases in Wound Healing

**Figure 1:**
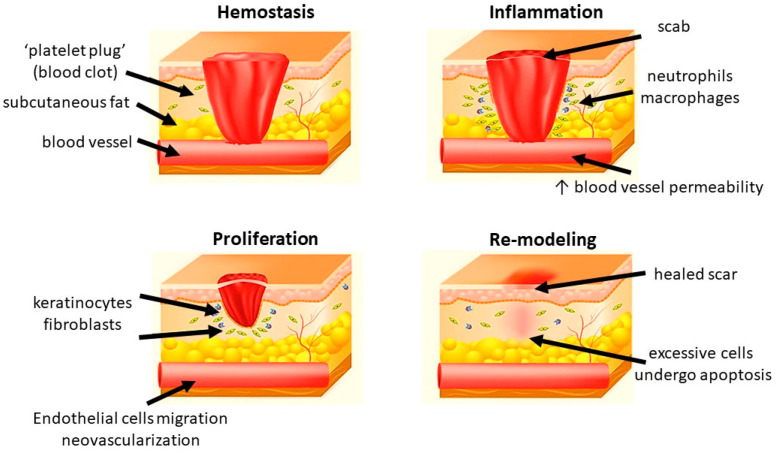
Different phases of wound healing.

Generally, wound healing consists of four highly integrated and overlapping physiological phases, including hemostasis, inflammation, proliferation, and re-modeling. They occur in the well-organized sequence and time frame [12]. Hemostasis is activated once the wound occurs to prevent blood loss. Subsequently, inflammatory response is activated to eliminate the foreign pathogens and to get the tissue prepare for restoration. In the proliferative phase, neovascularization, fibroblast migration, and re-epithelialization take place in a proper manner. Finally, the granulation tissue is replaced by a scar in the re-modeling phase [13,14,15] (Figure 1).

#### 1.2.1. Hemostasis

Hemostasis happens shortly after a wound has been formed (up to 2–4 h). Platelets reach to the wound first and form a clot to stop bleeding in a mechanical manner. After the initial “platelet plug” is formed at the site of injury, platelet-derived growth factor (PDGF) and platelet-derived SDF-1α are secreted. They contribute to the regulation of smooth muscle cells (SMCs) and bone marrow-derived endothelial progenitor cells, resulting in chemotaxis and proliferation of endothelial cells [16,17]. The clot also releases some cytokines, including transforming growth factor (TGF) β1, which promotes inflammatory cells proliferation and initiate the inflammatory phase. In addition to platelets, various components of blood, including certain coagulation factors and the fibrinolytic system, also play a role in this phase [18]. Additionally, vasoconstriction leads to local hypoxia, which triggers the following oxidative stress-related cascades.

#### 1.2.2. Inflammation

Inflammation is the body reaction against structural and functional impairments. It could be caused by mechanical forces, pathogens, toxins, and invasion of micro-organisms [19]. Inflammation occurs soon after hemostasis (immediate up to 2–5 days) and commonly lasts for 72 h [20]. After the vasoconstriction induced by hemostasis, the presence of histamine and other vasodilators increase the vascular permeability and therefore promote the migration of neutrophils and monocytes [21]. Neutrophils migrate to the injury site, kill the bacteria, and clear cellular debris through generation of free radicals, secretion of proteolytic enzymes, and phagocytosis [22,23]. Another important regulatory cell in the inflammatory phase is macrophage which is derived from monocytes. They remove non-functional host cells, bacterial-filled neutrophils, damaged matrix, foreign debris, and remaining bacteria through phagocytic effect [24]. Furthermore, the activated macrophages secret numerous growth factors, cytokines, and chemokines, including TGF-α, TGF-β, basic fibroblast growth factor (bFGF), PDGF, vascular endothelial growth factor (VEGF), and reactive oxygen species (ROS). They contribute to resolve inflammation, recruit endothelial cells and fibroblasts to active the proliferative phase. After this step, the levels of pro-inflammatory cytokines and oxidative stress gradually return to a basal state [25]. In chronic wound, the healing process is stuck in the inflammatory phase, leading to pro-longed hypoxia and excessive scaring.

#### 1.2.3. Proliferation

In this phase, epithelial proliferation is stimulated by epidermal growth factor (EGF) and TGF-α secreted by the activated platelets and macrophages [26]. Keratinocytes are critical in the process. After injury, they will migrate across the wound bed and anchor to the cell basement membrane. The migration is activated by keratinocyte growth factor (KGF)-1, KGF-2 and IL-6 secreted by fibroblasts [27,28]. Once the migration completes, the keratinocytes settle, proliferate, and differentiate into epidermis [29]. Angiogenesis is another important factor in scar formation. It is marked by endothelial cell migration and neovascularization. Endothelial cells are activated by vascular endothelial growth factor (VEGF), which is secreted by keratinocytes, macrophages, fibroblasts, and platelets. They migrate from healthy vessels and pass through the extracellular matrix (ECM) to the injury site by producing degradation factors, such as plasminogen activator and collagenase. Finally, they form new blood vessels and join the capillary loops, resulting in the provision of new blood supply to the wound. Hypoxia is reduced and ROS level decreases. After the cells are fully perfused, the excessive blood vessels undergo apoptosis [30,31]. Fibroblasts are essential in granulation tissue formation. They start to migrate from the wound margin in the inflammatory phase. With the stimulation of bFGF, TGF-β, and PDGF, they proliferate and synthesize glycosaminoglycans, proteoglycans, elastin, and fibronectin to form the new extracellular matrix of granulation tissue and collagen [32]. Collagen deposition provides the tensile strength to the wound and contributes to the cell migration, proliferation, and differentiation as a feeder layer [33]. The excessive collagen will be degraded by collagenases [34].

#### 1.2.4. Re-Modeling

Maturation and re-modeling are the final steps of wound healing. Clinically, re-modeling is the most important phase for patients. It is marked by the deposition and re-organization of collagen into the normal network [35]. An abnormal re-modeling will compromise the wound strength or might form a hypertrophic scar. The whole phase may last for weeks to years. In this phase, collagen III is degraded by collagenases and replaced by collagen I [36]. The new fibers are well-arranged into network. Meanwhile, macrophages, keratinocytes, fibroblasts, and myofibroblasts undergo apoptosis to reduce the excessive cells. The fibroblasts and matrix metallopeptidases (MMPs) regulate the ECM synthesis and cell differentiation, respectively. Taken all the above factors together, the tensile strength of the wound is enhanced, and a healed scar finally appears [17,37]. Meanwhile, ROS return to a physiological low level and hypoxia is abolished [25].

## 2. Conventional Methods in Wound Management

The standard of wound care includes debridement, cleaning, infection management, and dressing [38]. Debridement aims to remove the non-viable tissue and expose healthy, well-perfused tissue through surgical or autolytic/enzymatic approaches [39]. After debridement, the wound can be cleaned using normal saline or sterile water [40]. Detergents, hydrogen peroxide, and concentrated povidone-iodine solution are not suggested due to tissue damage and their toxicity [41]. The management of infection is critical in wound healing. Failure in infection control leads to poor healing and abscess formation. Both topical and systemic antimicrobials are used for the infected wounds. Topical agents are commonly used for superficial wound infection, while systemic antibiotics are applied in patients with deep or systemic infection [42]. Different types of dressing have been developed to protect the wound from infection and to promote the healing process. Dry gauze is a traditionally used wound dressing, but it may cause secondary injury during removal. Moisture-retentive dressings (MRDs) are materials with moisture vapor transmission rates (MVTRs) less than 35 g/m^2^/h, which allow the wound to heal in a moist environment. There are five basic types of MRDs, including films, foams, hydrocolloids, alginates, and hydrogels. Some of them are adherent and absorbent. Some are not and need a secondary dressing to keep in place [43].

Skin substitutes encompass a diverse range of biological, synthetic, or biosynthetic materials that can effectively provide either temporary or permanent coverage for open skin wounds. These substitutes are valuable in the treatment of both acute and chronic wounds, serving to cover defects resulting from burns or other injuries, as well as for reconstructive purposes, such as in the release of extensive post-burn contractures. They have been used in patients with wounds for a long time. The split-thickness autograft is widely used in the chronic wound. The efficiency largely depends on the quality and quantity of donor skin. The pain and donor site infection also limit the approach [44]. Skin substrates refer to the underlying tissue supporting the skin. The bio-engineered skin substrates are a kind of dressing, which mimic the architecture of normal skin and promote wound healing in patients. They are categorized into dermal, epidermal, and full skin. According to the biologic source, the substrates can be autogenic (host of the transplant), allogenic (another human donor) or xenogenic (another species) [45].

There are some traditional approaches, such as plant extracts and herbal medicines, used for promoting wound healing. Aloe vera is widely used in primary care. It has various biological and pharmacological activities including antioxidant, anti-inflammatory, immuno-modulatory, antimicrobial, and skin-protective [46,47]. NF3 is an innovative herbal formula developed by our institute. It consists of two herbs: Astragali Radix and Rehmanniae Radix in the ratio of 2:1 (*w*/*w*). The formula enhanced the diabetic ulcer healing through promoting angiogenesis and suppressing inflammation via in vitro, in vivo, and clinical studies [48,49,50].

## 3. Modern Approaches in Wound Healing

**Figure 2:**
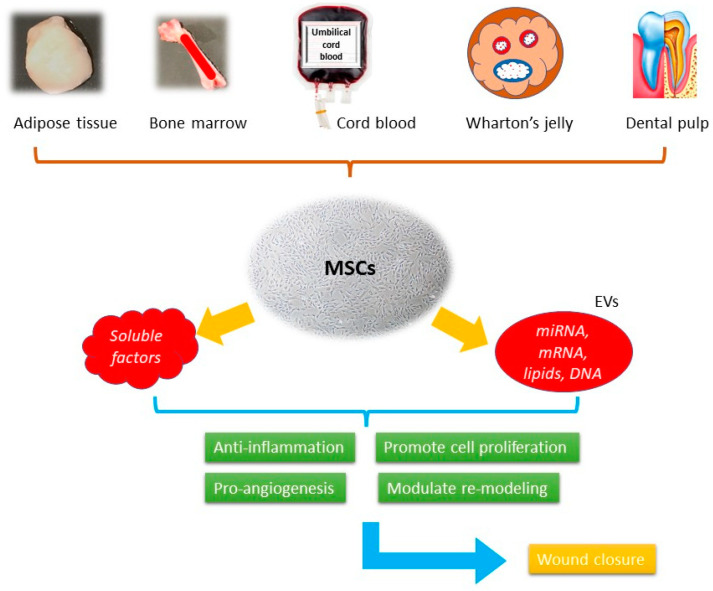
The schematic picture of the mechanisms of MSCs-based cell-free therapy in wound healing.

In modern therapeutic practice, the use of stem cells is becoming a promising candidate. They present with a high self-renewing capacity and various linage differentiation ability. In wound healing, they could overcome the limitations of traditional approaches, such as contamination and tissue irritation, while promoting tissue regeneration (Figure 2).

### 3.1. Overviews of Mesenchymal Stem Cells (MSCs)

Mesenchymal stem cells (MSCs) were first isolated from bone marrow in the 1970s, being the progenitor cells coming from mesodermal origin [51], they could differentiate into mesenchymal lineages including osteoblasts, chondrocytes, myocytes, and adipocytes, but not hematopoietic stem cells. MSCs can be isolated from different tissues such as bone marrow, adipose tissue, peripheral blood, umbilical cords, Wharton’s jelly, and dental pulp [52] (Figure 1). According to the report published by International Society for Cellular Therapy (ISCT), when cultured in a standard condition (37 °C, 5% CO_2_, atmospheric O_2_ concentration (~20%) in a humidified incubator with media containing 10% fetal bovine serum) the minimum inclusion criteria for defining MSCs include: (i) The ability to adhere to the plastic bottom under standard culture condition; (ii) the presence of specific surface markers, such as CD73, CD105, and CD90 as well as the absence of a series of surface markers, such as CD14, CD19, CD34, and CD45; (iii) the ability to differentiate into osteoblasts, chondrocytes, and adipocytes in vitro under the effect of specific culture media [53]. They demonstrate the therapeutic effects through immune-modulation, anti-inflammation, pro-angiogenesis, anti-oxidative, and anti-apoptotic activities [54]. These effects are presented through paracrine activities rather than direct cell differentiation [55]. The secretome, or the conditioned medium (CM), is a composite product secreted by MSCs. It consists of soluble proteins (mostly are growth factors, cytokine, and chemokines) and extracellular vesicles (EVs), where proteins, lipids, and genetic materials are encapsulated and transferred. Despite the availability of numerous skin substitutes in the market, stem cells are considered a better option for wound healing. They have been shown to promote healing in various types of wounds, including acute wound, chronic wound, and burn wound. It involves the use of living cells, which have the ability to actively interact with the wound environment and promote healing through the secretion of growth factors and other signaling molecules. This approach has been shown to be effective in treating chronic wounds that do not respond well to conventional therapies. When using cell-free products, such as growth factors, cytokines, and extracellular vesicles, it can stimulate the body’s natural healing mechanisms without the need for live cells [56]. In contrast, many skin substitutes in the market are often composed of synthetic materials that lack the biological complexity and versatility of living cells.

### 3.2. Soluble Factors in Mesenchymal Stem Cells (MSCs) Derived Secretome

A growing number of studies demonstrated MSCs could secrete a panel of trophic factors, including cytokines, growth factors, and chemokines. The activity of the secretion, which was first described in 2006, makes MSCs a paracrine tool [56,57]. These trophic factors could contribute to different wound-healing phases (Table 1).

#### 3.2.1. Inflammation with Influence of MSCs’ Soluble Factors

**Figure 3:**
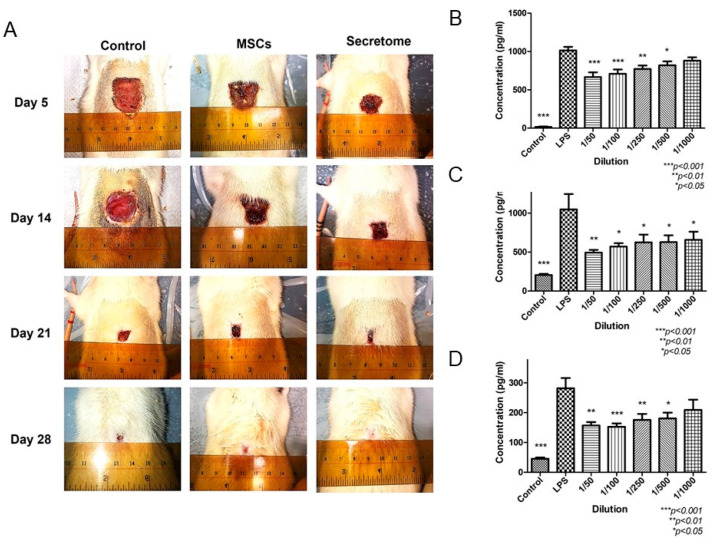
MSCs-derived secretome promoted wound healing in full thickness excision model through anti-inflammatory activity [58]. (**A**): Photos of wound at different time points. Both MSCs and secretome promoted wound healing, and rats with secretome application showed complete healing on day 28. (**B**–**D**): ELISA result of pro-inflammatory cytokines IL-6, MCP-1 and TNF-α, respectively. After treated by different doses of secretome, the level of pro-inflammatory cytokines was reduced.

The inflammatory phase is critical in wound healing. The impairment in this phase leads to chronic non-healing ulcer. The switch of the macrophages’ phenotype from M1 pro-inflammatory to M2 anti-inflammatory is the key step to control inflammation. There are several cytokines secreted by MSCs, which regulate the polarization of macrophages. TGF-β is found in MSCs-derived secretome, together with IL-1β and IL-6, promoted macrophage recruitment and polarization from pro-inflammatory phenotype (M1) to anti-inflammatory phenotype (M2) [59]. Our previous study demonstrated MSCs-derived secretome suppressed the release of IL-6, TNF-α, and MCP-1 from M1 macrophage (Figure 3) [58]. Another study demonstrated that CM from bone marrow-derived stem cells reduced the inflammatory cells (neutrophils and macrophages) infiltration in vivo at various time points [60]. A group of proteins related to inflammation have been identified in MSCs-derived secretome (Table 1) [59,60,61,62,63,64].

#### 3.2.2. Proliferation and Angiogenesis with Influence of MSCs’ Soluble Factors

**Figure 4:**
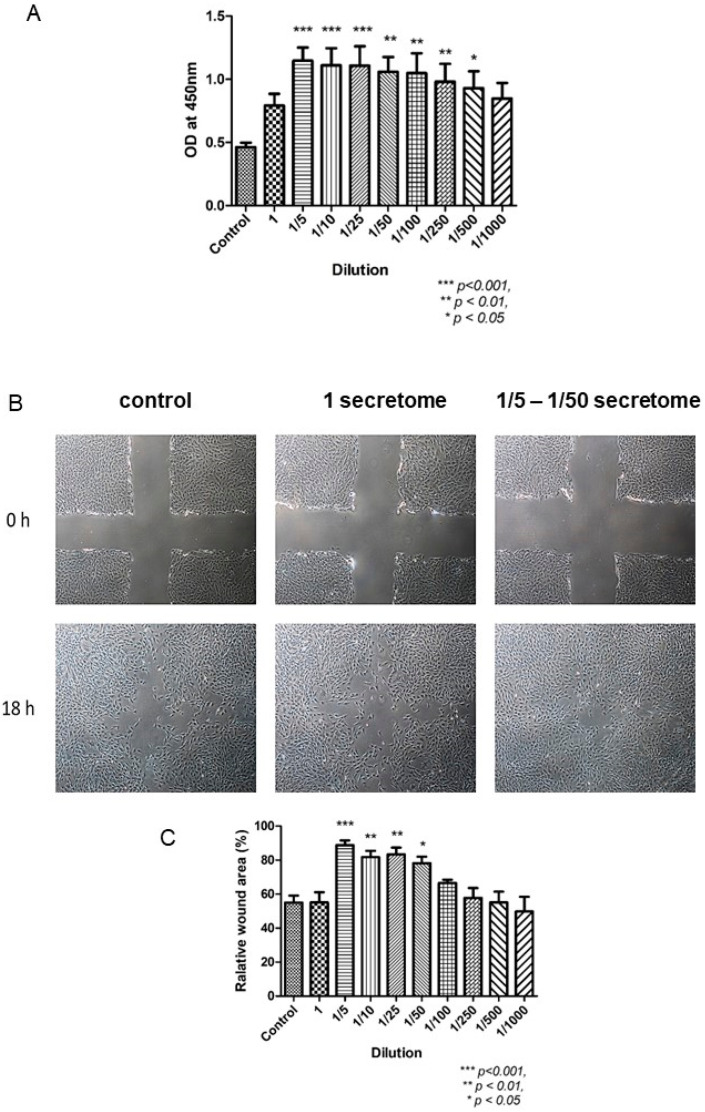
The effects of MSCs-derived secretome in fibroblasts [58]. (**A**): BrdU proliferation assay indicated the proliferation ability of FR was enhanced when treated with various concentration of secretome. (**B**): Photos of scratch test taken at 0 h and 18 h. After treated with different concentrations of secretome for 18 h, the wound was closed at various degrees. (**C**): Statistical results indicated that the wound area was reduced significantly.

In the proliferative phase, the wound must be filled and covered in a proper manner. Fibroblasts and epithelial cells play an important role in the contraction of wound margin. The application of MSCs-derived secretome enhanced the proliferation and migration of fibroblasts and increased ECM deposition, contributing to wound healing (Figure 4) [59]. Besides anti-inflammation as discussed above, TGF-β is also essential in the proliferative phase. It could increase the local ECM secretion, regulate melanin production, activate keratinocyte differentiation and maturation [57]. It was reported that TGF-β, together with MMP-9, promoted wound healing in diabetic ulcer [57,65]. The presence of TGF-β in MSCs-derived secretome has been confirmed (Table 1).

MSCs secrete a large group of angiogenic factors, including bFGF, TGF-β, PDGF, VEGF, HGF, and MCP-1, which promote neovascularization both in vitro and in vivo (Table 1). HGF presents the angiogenetic property by increasing the expression of VEGF. VEGF and TGF-β then function through the PI3K/Akt and MAPK pathways [66]. The angiogenic effects of MSCs-derived secretome may differ due to different origins (Wharton’s Jelly (WJ), adult tissues, bone marrow, neonatal tissues). It was reported that compared with bone marrow-derived MSCs, MSCs derived from adult tissue released higher level of angiogenic factors, such as VEGF, insulin-like growth factor 1 (IGF-1), IL-8, and MMP3, exhibited a stronger pro-angiogenetic ability [67]. However, the conclusion remained controversial. In a recent study, the angiogenic factors released from three different tissues (bone marrow, Wharton’s Jelly, and adult tissue) were compared using proteomic analysis. The concentration of the angiogenic profiles was higher in the CM of the MSCs derived from bone marrow and WJ than that derived from adult tissue, due to a higher expression of AKT serine/threonine kinase 1 (AKT1) and bFGF [66]. The reasons could be the variations among different samples and the lack of a standardized process. Hence, a common and standardized protocol for secretome collection is being investigated for years. Furthermore, the localized hypoxia present in the wound area helps regulate certain transcription factors, notably hypoxia-inducible factor (HIF), which plays a critical role in the regulation of VEGF. Research studies suggest that hypoxic preconditioning can modify the profile of CM from MSCs, ultimately boosting their ability in angiogenesis [25,64].

#### 3.2.3. Re-Modeling with Influence of MSCs’ Soluble Factors

Re-modeling is the last step in wound healing. It is characterized by collagen synthesis and maturation. Together with the previous steps, the skin at the injury site regains strength and flexibility. If the re-modeling phase is retarded, the tissue will be left with scar or will remain in chronic non-healing state. The secretome derived from mesenchymal stem cells (MSCs) contains various cytokines and growth factors, including hepatocyte growth factor (HGF) and prostaglandin E2 (PGE2). These factors possess anti-fibrotic properties by inhibiting myofibroblast differentiation and preventing epithelial-mesenchymal transition (Table 1) [68,69]. Furthermore, the MSC-derived secretome stimulates the proliferation, migration, and differentiation of local progenitor cells into new keratinocytes, which is essential for tissue remodeling. Additionally, the secretome can modulate the behavior of other cells, including endothelial cells and fibroblasts, by promoting cell survival and enhancing extracellular matrix production [70].

### 3.3. Extracellular Vesicles (EVs) in Mesenchymal Stem Cells (MSCs) Derived Secretome

Extracellular vesicles (EVs) carry mRNA, mircro RNA, and proteins to modulate intercellular communications. They can be found in almost all biological fluids. Based on the size of EVs, they could be categorized into three fractions: exosomes (Exos), microvesicles (MVs), and apoptotic bodies [71]. Exosomes (Exos), with the size of 20 and 150 nm, are formed within the endosomal space. They are released into the extracellular area after the fusion with the multivesicular bodies of the plasma membrane. Exos were first reported in 1983 as vesicles contributing to mammalian reticulocyte differentiation and maturation [72]. In the recent research, they were found to be similar to MSCs, and played an important role in cell–cell communication [73]. Microvesicles (MVs), with a larger size of 100 to 1000 nm, are formed by direct budding of the plasma membrane. Apoptotic bodies, with a diameter between 50 nm and 5 µm, are secreted by membrane blebbing as programmed cell death occurs [74]. Since the size of different fractions could be overlapping, the general term “extracellular vesicles (EVs)” will be used in the following section. The International Society for Extracellular Vesicles (ISEV) has published the latest Minimal Information for Studies of Extracellular Vesicles (MISEV) guidelines, which outline the criteria that should be followed for each preparation of EVs. These criteria include quantitative measures of the source of EVs (such as the number of secreting cells, volume of biofluid, or mass of tissue), determination of EV abundance (total particle number and/or protein or lipid content), identification of components associated with EV subtypes or EVs generically, and identification of any non-vesicular, co-isolated components [74]. Differential ultracentrifugation is the most commonly used technique for separating EVs. However, other chemical-based techniques, such as density gradients, precipitation, and immune-isolation, are also available. The choice of EV isolation method depends on various factors, including the desired recovery rate, specificity, and purity. The therapeutic effects of MSCs-derived EVs are related to their cargo, including microRNA, mRNA, and proteins, and have been demonstrated in the wound models (Table 2) [75,76,77,78,79,80,81,82,83,84,85,86,87,88,89,90,91].

#### 3.3.1. EVs’ Influence in Inflammation

Inflammation is essential in triggering tissue regeneration as discussed above (Section 1.2.2). Prolonged and uncontrolled inflammatory response leads to chronic diseased conditions. EVs are reported to present similar immune-modulation properties as MSCs from which they are secreted. They suppress T-cells proliferation and activate T cells into T regulatory cells [76]. Meanwhile, they induce the macrophage to present M2 anti-inflammatory phenotype and reduce the level of pro-inflammatory factors including inducible nitric oxide synthase (iNOS), IL-1β, cyclooxygenase-2 (COX-2), and MCP-1 [77,78]. Despite the similar mechanisms they present, MSCs and EVs regulate the inflammation through different routines. The anti-inflammatory effect of EVs is mainly related to their cargo “microRNA content” [78]. miR-223, which was derived from human BM-MSC-EVs, regulated macrophage M2 polarization, reduced the expression of TNF-α, up-regulated IL-10 expression, and promoted cutaneous wound healing by targeting pknox1 [79]. In a diabetic wound model, LPS-pretreated human umbilical cord (UC)-MSCs-EVs promoted M2 macrophage polarization, reduced inflammatory response and accelerated wound healing by miR-let7b through the inhibition of the TLR4/NF-kB pathway and STAT3/AKT pathways [80]. Some other miRNAs in EVs, such as miR-181c, miR-let7, miR-182, and miR-155, have been reported to show immuno-modulatory property (Table 2) [81,82].

#### 3.3.2. EVs’ Influence in Proliferation and Angiogenesis

MSCs-derived EVs stimulated the proliferation and migration of skin cells, including fibroblasts and keratinocytes which are the essential cell types in the proliferative phase. MSC-EVs enhanced the proliferation and migration of fibroblasts harvested from both healthy and chronic wound donors, activated various signaling pathways, including Akt, ERK, and STAT3. They also stimulated the expression of a series of growth factors such as HGF, IGF-1, and nerve growth factor (NGF) [83]. Wnt-4 was reported to be contained in MSCs-EVs, which promoted β-catenin nuclear translocation and enhanced proliferation and migration of skin cells. Moreover, they activated AKT pathway and reduced heat stress-induced apoptosis in rat skin burn model [84]. Some studies demonstrated that the miRNAs transferred to the target cells were critical for the therapeutic effects. miR-21 was found highly expressed in MSCs-EVs. It enhanced the migration and proliferation of HaCaT (keratinocytes) cells by modulating the expressions of MMP-2 and TIMP-1, targeting the PI3K/AKT signal pathway [85]. The functions of other miRNAs, including miR-378 and miR-19b, were also reported in the wound healing models (Table 2) [86,87,88].

In terms of neovascularization, MSCs-EVs also presented the pro-angiogenic effect. They were shown to enhance the proliferation, migration of fibroblasts and endothelial cells, and up-regulated the expression of angiogenesis-related growth factors in endothelial cells including PDGF, VEGF, FGF, and HIF-α [89]. The in vivo angiogenic property of MSC-EVs was investigated under blue light illumination using both murine Matrigel plug and skin wound models [90]. The proliferation and migration of endothelial cells was activated. The miR-135b-5p and miR-499a-3p in MSC-EVs were found up-regulated in response of the activation (Table 2) [90]. In a separate study, EVs were extracted from BMSCs that were pre-treated with deferoxamine, a hypoxia-mimicking agent known to reduce iron-mediated ROS production and stabilize HIF-1α, thereby promoting tissue vascularization. These EVs were found to contain miR-126, which was identified as a stimulator of angiogenesis through the PI3K/AKT signaling pathway (Table 2) [91,92].

#### 3.3.3. EVs’ Influence in Re-Modeling

Matrix re-modeling is the last step in wound healing, marked by collagen deposition and ECM re-organization. In the study using BMSC-EVs, the collagen deposition was increased significantly at wound area [79]. The control of myofibroblasts differentiation and collagen I deposition are critical in regulating ECM re-modeling. miR-21-5p and miR-125b-5p, which were identified in umbilical cord blood (UCB)-MSC-EVs, targeted the TGF-β signaling pathway, inhibited TGFBR1 and TGFBR2, as well as suppressed human dermal fibroblast (HDF) differentiation into myofibroblasts [93]. Similarly, the anti-fibrotic effect of miR-29a-3p was investigated in EVs from human ASC-MSCs [94]. It targeted Dnmt3a, Pdgfrbb, Bcl2, Bcl-xl and modulated skin fibrosis through the regulation of methylation and apoptosis [94].

### 3.4. Clinical Trials Using MSCs-Based Cell-Free Products

The therapeutic effects of MSC-derived secretome have been demonstrated in various disease models. Nevertheless, their clinical application remains limited. According to the records on a database of funded clinical studies conducted around the world (www.clinicaltrials.gov (accessed on 13 March 2023), there is one completed phase I clinical trial registered, in which conditioned medium from WJ-MSC has been used for chronic ulcer (NCT04134676). A total of 38 patients with chronic ulcer for more than one month were enrolled, and topical conditioned medium gel covered by transparent dressing was applied for two weeks. The evaluation and dressing replacement were performed once a week. The primary results suggested that edema and erythema were reduced, granulation tissue presented, and the size of ulcer reduced (Table 3). In terms of exosome, there is also one completed phase I clinical trial registered (NCT02565264). About 5 participants were enrolled. Autologous plasma-derived exosome was applied to the ulcer daily for 28 days. Ulcer size and pain level were recorded once a week. Unfortunately, neither results nor related publications have been uploaded by the investigators so far (Table 3).

Except for wound management, there are some clinical trials registered using MSC-based cell free products for other diseases, such as Alzheimer’s disease (NCT04388982), stroke (NCT05008588), alopecia (NCT05658094, NCT05296863), and dry eye diseases (NCT04213248, NCT05738629). Since the outbreak of severe acute respiratory syndrome coronavirus 2 (SARS-CoV-2) in 2019, a great number of clinical trials have been registered using MSCs-derived CM (secretome) or exosomes against the respiratory symptoms (Table 3).

### 3.5. Hopes and Challenges

#### 3.5.1. Advantages of MSC-Based Cell-Free Therapy

Although the use the cell-based products to promote wound healing is not new, there are some barriers to overcome. Most of the commercial cell-based skin graft substitutes, such as Apligraf^®^, Recell^®^, PolyActive^®^, and OrCel^®^, are expensive [95]. They require special and strict conditions for storage and transportation. Furthermore, there are potential risks, including tumorgenicity, rejection, and infection, make them difficult to be used commonly. The delivery of living cells to the wound site is another challenge. The injection of cells through a needle will damage cell membrane and viability [96]. After injection, the excessive apoptotic or necrotic cells may elicit the local immune response, leading to a secondary impairment. Hence, different acellular approaches are needed.

Numerous preclinical studies have demonstrated the therapeutic effects of MSCs-derived cell-free products (MSCs-CM). Different animal models were used to investigate their efficacy. The in vitro experiments contributed to a better understanding of the underlying mechanisms. Compared with the cell-based products, the MSCs-CM can be manufactured, packaged, stored, and transported more easily. Large-scale production is therefore possible. Treated as pharmacological agents, the quality control of the cell-free products is simpler. There is no need to match the donor and the recipient and can avoid the immune rejection. Furthermore, they can reduce the possibility of emboli formation, tumorgenicity, and infection transmission [97]. Therefore, unlike cell-based products, MSCs-CM can be regarded as a ready-to-use pharmacological agent. In general, isolated cell-free products can be stored at temperatures ranging from −20 °C to −80 °C for up to 6 to 7 months [98].

#### 3.5.2. Constraints and Challenges in Cell-Free Products

Although the effectiveness of MSC-based cell-free products have been demonstrated in many preclinical studies, there are still limited clinical research and commercial products. The first challenge is the profiling of the components. In addition to the soluble factors, there are different EVs in secretome, containing miRNAs, lipids, and long noncoding RNAs. Their identification and bioactivities are not completely understood. The comprehensive and quantitative analysis of the biomolecules in secretome using “omics” approaches is needed to clarify their profile and functions. The lack of a standardized isolation and cultivation protocol is the second challenge in the field. The difference in donors (gender, age, tissue source), cell passage and numbers (seeding density), laboratory conditions (medium, flask, oxygen tension), and preparation procedure (particularly ultracentrifugation versus precipitation for EVs) lead to the variation in the components of secretome from batch to batch. As an illustration, the secretome profile of MSCs derived from varying tissue sources can differ, while the composition of the medium and culture supplements used can affect cell viability and proliferative capacity. Additionally, studies have revealed that subjecting cells to lower oxygen tension (below 5%) can enhance the regenerative potential of the secretome, particularly in the context of ischemic diseases. The production of MSC-secretome using pharmacological standard could reduce the inconsistency. Compliance with a good manufacturing practice (GMP) standard protocol is also necessary to translate the cell-free products bench to bed [63,99]. Up to today, there are not enough comprehensive studies focusing on the different application protocols for secretome, such as preparation methods, concentrations, and delivery routes. A specific protocol may be needed for a given pathology. Meanwhile, in most reported studies, fetal bovine serum (FBS) was used for MSCs’ cultivation. It could be unsuitable for clinical use due to possible disease transmission and xenogeneic immune reaction [100]. Therefore, human serum or knockout serum replacement is needed, which will largely increase the cost of the cell-free products as expected [101].

## 4. Conclusions

MSCs-based cell-free products have emerged as a new and promising therapeutic option in tissue regeneration. They bypass the risks of tumorgenicity, immune rejection, and ethical issues in cell therapy. A growing number of preclinical studies have demonstrated the therapeutic effects of MSCs secretome, including the soluble factors and EVs. However, the number of clinical trials remains limited. To get a large-scale clinical grade cell-free product, a well-defined and standardized manufacturing protocol is needed. The experiences in developing GMP compliant pharmacological agents can be applied. Despite the incomplete knowledge about the underlying mechanisms, the cell-free therapy provides an additional therapeutic option for patients suffering from chronic non-healing wound.

## Figures and Tables

**Table 1:** Proteins related to wound healing in MSCs-derived secretome [58,59,60,61,62,63,64].

Proteins	Inflammation	Proliferation	Angiogenesis	Re-Modeling	Ref.
ANG-1			✓		[61]
BMP		✓			[64]
CCL2	✓				[62]
CCL5	✓				[64]
CCL20	✓				[62]
EGF		✓	✓		[63,64]
FGF-7		✓		✓	[60,61,64]
Follistatin				✓	[61]
G-CSF		✓			[64]
GM-CSF		✓			[64]
HGF		✓	✓	✓	[60,61,63,64]
ICAM				✓	[62]
IDO	✓				[64]
IGF		✓	✓		[63,64]
IL-6	✓		✓	✓	[60,61,63,64]
IL-8	✓				[63,64]
IL-10	✓			✓	[64]
Insulin			✓		[61]
LIF	✓				[62,64]
Lipocalin-2	✓				[62]
MCP-1	✓		✓		[60,63,64]
MMP-1			✓	✓	[60,64]
MMP-2	✓		✓		[62,64]
MMP-3			✓	✓	[62,64]
MMP-7			✓	✓	[64]
PDGF		✓	✓		[63,64]
PGE2	✓	✓			[64]
Serpin E1				✓	[62]
TGF-β1	✓	✓	✓	✓	[61,63]
TIMP-1				✓	[60,64]
TIMP-2				✓	[62,64]
uPAR			✓	✓	[61]
VEGF			✓		[58,60,61,62,63,64]

**Table 2:** Contents identified in MSCs-EVs related to wound healing [76,77,78,79,80,81,82,83,84,85,86,87,88,89,90,91].

Inflammation Refs. [76,77,78,79,80,81,82]	Proliferation Refs. [84,85,86,87,88]	Angiogenesis Refs. [90,91]	Re-modeling Refs. [92,93]
miR-223, miR-let7b, miR-181c, miR-let7, miR-182, miR-155	Wnt-4, miR-21, miR-378, miR-19b, MALAT1	miR-135b-5p, miR-499a-3p, miR-126	Wnt-4, miR-21, miR-23a, miR-125b, miR-145, miR-29a-3p

**Table 3:** Clinical trials using MSC-based cell-free products registered in ClinicalTrials.gov (13 Mar 2023).

Disease	Cell-Free Products	Study Phase	Status	ClinicalTrials.gov Identifier
Skin disease				
Wound	AD-MSC-exo	N.A.	Not yet recruiting	NCT05475418
	Plasma-exo	Phase 1	Unknown	NCT02565264
	WJ-MSC-CM	Phase 1	Completed	NCT04134676
	MSC-CM	Phase 1	Unknown	NCT04235296
Psoriasis	Commercial MSC-exo	Phase 1	Completed	NCT05523011
Dystrophic Epidermolysis Bullosa	Commercial MSC-EVs	Phase 1/2A	Not yet recruiting	NCT04173650
Skin aging	AD-MSC-CM	N.A.	Completed	NCT05508191
Hypertrophic scar	MSC-CM	N.A.	Not yet recruiting	NCT05004779
Keloid	UC-MSC-CM	Phase 1/2	Unknown	NCT04326959
Respiratory disease				
COVID-19	MSC-exo	Phase 1	Completed	NCT04276987
	MSC-exo	Phase 2/3	Recruiting	NCT05216562
	BM-EVs	Phase 2	Completed	NCT04493242
	MSC-exo	Phase 1/2	Completed	NCT04491240
	Commercial MSC-exo	Phase 1/2	Not yet recruiting	NCT04798716
	Commercial WJ-MSC-exo, UC-MSC-exo	Phase 1	Recruiting	NCT05387278
	MSC-CM	Phase 3	Completed	NCT05122234
	MSC-CM	Phase 2	Recruiting	NCT04753476
Pulmonary infection	AD-MSC-exo	Phase 1/2	Recruiting	NCT04544215
Acute lung injury	Body fluid-exo	N.A.	Recruiting	NCT05058768
ARDS	hMSC-exo	Phase 1/2	Unknown	NCT04602104
Neurological disease				
Alzheimer’s disease	AD-MSC-exo	Phase 1/2	Unknown	NCT04388982
Ischemic stroke	MSC-exo	Phase 1/2	Unknown	NCT03384433
Cerebral palsy	UC-MSC-CM	Phase 1/2	Recruiting	NCT04314687
Stroke	UC-MSC-CM	Phase 1/2	Recruiting	NCT05008588
Orthopedic disease				
Osteoarthritis	MSC-EVs	Phase 1	Not yet recruiting	NCT05060107
	AD-MSC-CM	N.A.	Recruiting	NCT04223622
	UC-MSC-CM	Phase 1/2	Recruiting	NCT04314661
	UC-MSC-CM	Phase 1/2	Active	NCT05579665
Degenerative Meniscal Injury	Synovial Fluid-MSC-exo	Phase 2	Recruiting	NCT05261360
Bone graft	AD-MSC-CM	Phase 1	Not yet recruiting	NCT04998058
Ligament injury	AD-MSC-CM	Phase 1	Recruiting	NCT04889963
Eye disease				
Macular holes	MSC-exo	Phase 1	Active	NCT03437759
Dry eye	UC-MSC-exo	Phase 1/2	Recruiting	NCT04213248
	PSC-MSC-exo	Phase 1/2	Not yet recruiting	NCT05738629
Retinitis pigmentosa	WJ-MSC-exo	Phase 2/3	Recruiting	NCT05413148
Ocular surface disease	MSC-CM	Phase 1	Not yet recruiting	NCT05204329
Other diseases				
Alopecia	Amniotic-MSC-exo	N.A.	Recruiting	NCT05658094
	AD-MSC-CM	Phase 3	Completed	NCT05296863
Otitis Media	Exosomes	N.A.	Not yet recruiting	NCT05402267
Atrial fibrillation	Epicardial fat-exo	N.A.	Recruiting	NCT03478410
Perianal Fistula	Placental-MSC-exo	Phase 1/2	Active	NCT05499156
T1DM	UCB-MSC-EVs	Phase 2/3	Unknown	NCT02138331
PCOS	UC-MSC-CM	Phase 1/2	Recruiting	NCT05279768
Nasopharyngeal cancer	Dendritic-CM	Phase 1/2	Recruiting	NCT05261750

AD-MSC: adipose-derived mesenchymal stem cells; Exo: exosomes; WJ-MSC: Wharton’s jelly derived mesenchymal stem cells; UC-MSC: umbilical cord-derived mesenchymal stem cells; MSC-CM: mesenchymal stem cells-derived conditioned medium; MSC-EVs: mesenchymal stem cells-derived extracellular vesicles; BM-EVs: bone merrow-derived extracellular vesicles; PSC-MSC: pluripotent stem cell-derived mesenchymal stem cells; UCB-MSC: umbilical cord blood-derived mesenchymal stem cells; ARDS: acute respiratory distress syndrome; T1DM: type I diabetes mellitus; PCOS: polycystic ovary syndrome.

## Data Availability

Not applicable.

## Abbreviations

ANG-1	angiopoietin-1
ARDS	acute respiratory distress syndrome
bFGF	basic fibroblast growth factor
BM	bone marrow
CCL	chemokine (C-C motif) ligand
CM	conditioned medium
COX2	cyclooxygenase 2
ECM	extracellular matrix
EGF	epidermal growth factor
EVs	extracellular vesicles
Exos	exosome
FBS	fetal bovine serum
G-CSF	granulocyte colony-stimulating factor
GM-CSF	granulocyte macrophage-colony stimulating factor
GMP	good manufacturing protocols
HDF	human dermal fibroblast
HGF	hepatocyte growth factor
HIF-1α	hypoxia-inducible factor 1-alpha
ICAM	intercellular adhesion molecule
IDO	indoleamine-pyrrole 2,3-dioxygenase
IGF	insulin-like growth factors
lncRNAs	long non-coding RNAs
iNOS	inducible nitric oxide synthase
ISCT	International Society for Cellular Therapy
KGF	keratinocyte growth factor
LIF	leukemia inhibitory factor
MAPK	mitogen-activated protein kinase
MCP-1	monocyte chemoattractant protein-1
MI	myocardial infarction
miRNAs	micro-RNAs
MMPs	matrix metallopeptidases
MRDs	moisture-retentive dressings
MSC	mesenchymal stem cell
MVTRs	moisture vapor transmission rates
NGF	nerve growth factor
PCOS	polycystic ovary syndrome
PDGF	platelet-derived growth factor
PGE2	prostaglandin E2
SMCs	smooth muscle cells
T1DM	type I diabetes mellitus
TGF	transforming growth factor
TIMPs	tissue inhibitor of metalloproteinase
TNF-α	tumor necrosis factor alpha
UM	umbilical cord
uPAR	urokinase-type plasminogen activator receptor
VEGF	vascular endothelial growth factor
WJ	Wharton’s Jelly

## References

[1] Heng M.C. (2011). Wound healing in adult skin: Aiming for perfect regeneration. Int. J. Dermatol..

[2] Reinke J.M., Sorg H. (2012). Wound repair and regeneration. Eur. Surg. Res..

[3] Monavarian M., Kader S., Moeinzadeh S., Jabbari E. (2019). Regenerative Scar-Free Skin Wound Healing. Tissue Eng..

[4] Ozgok Kangal M.K., Regan J.P. (2022). Wound Healing. StatPearls.

[5] Karppinen S.M., Heljasvaara R., Gullberg D., Tasanen K., Pihlajaniemi T. (2019). Toward understanding scarless skin wound healing and pathological scarring. F1000Research.

[6] Raziyeva K., Kim Y., Zharkinbekov Z., Kassymbek K., Jimi S., Saparov A. (2021). Immunology of Acute and Chronic Wound Healing. Biomolecules.

[7] Korting H.C., Schöllmann C., White R.J. (2011). Management of minor acute cutaneous wounds: Importance of wound healing in a moist environment. J. Eur. Acad. Dermatol. Venereol..

[8] Grada A., Phillips T.J. (2022). Nutrition and cutaneous wound healing. Clin. Dermatol..

[9] Järbrink K., Ni G., Sönnergren H., Schmidtchen A., Pang C., Bajpai R., Car J. (2016). Prevalence and incidence of chronic wounds and related complications: A protocol for a systematic review. Syst. Rev..

[10] Zamboni P., Gemmati D. (2007). Clinical implications of gene polymorphisms in venous leg ulcer: A model in tissue injury and reparative process. Thromb. Haemost..

[11] Bowers S., Franco E. (2020). Chronic Wounds: Evaluation and Management. Am. Fam. Physician.

[12] Nagle S.M., Stevens K.A., Wilbraham S.C. (2022). Wound Assessment. StatPearls.

[13] Rodrigues M., Kosaric N., Bonham C.A., Gurtner G.C. (2019). Wound healing: A cellular perspective. Physiol. Rev..

[14] Tottoli E.M., Dorati R., Genta I., Chiesa E., Pisani S., Conti B. (2020). Skin wound healing process and new emerging technologies for skin wound care and regeneration. Pharmaceutics.

[15] Ellis S., Lin E.J., Tartar D. (2018). Immunology of wound healing. Curr. Dermatol. Rep..

[16] Golebiewska E.M., Poole A.W. (2015). Platelet secretion: From haemostasis to wound healing and beyond. Blood Rev..

[17] Wilkinson H.N., Hardman M.J. (2020). Wound healing: Cellular mechanisms and pathological outcomes. Open. Biol..

[18] Opneja A., Kapoor S., Stavrou E.X. (2019). Contribution of platelets, the coagulation and fibrinolytic systems to cutaneous wound healing. Thromb. Res..

[19] Sinno H., Prakash S. (2013). Complements and the wound healing cascade: An updated review. Plast. Surg. Int..

[20] Hassanshahi A., Moradzad M., Ghalamkari S., Fadaei M., Cowin A.J., Hassanshahi M. (2022). Macrophage-Mediated Inflammation in Skin Wound Healing. Cells.

[21] Silva J.R., Burger B., Kühl C.M.C., Candreva T., Dos Anjos M.B.P., Rodrigues H.G. (2018). Wound Healing and Omega-6 Fatty Acids: From Inflammation to Repair. Mediat. Inflamm..

[22] Zhu S., Yu Y., Ren Y., Xu L., Wang H., Ling X., Jin L., Hu Y., Zhang H., Miao C. (2021). The emerging roles of neutrophil extracellular traps in wound healing. Cell. Death Dis..

[23] Phillipson M., Kubes P. (2019). The Healing Power of Neutrophils. Trends Immunol..

[24] Louiselle A.E., Niemiec S.M., Zgheib C., Liechty K.W. (2021). Macrophage polarization and diabetic wound healing. Transl. Res. J. Lab. Clin. Med..

[25] Lopez T., Wendremaire M., Lagarde J., Duquet O., Alibert L., Paquette B., Garrido C., Lirussi F. (2022). Wound Healing versus Metastasis: Role of Oxidative Stress. Biomedicines.

[26] Zubair M., Ahmad J. (2019). Role of growth factors and cytokines in diabetic foot ulcer healing: A detailed review. Rev. Endocr. Metab. Disord..

[27] Rousselle P., Montmasson M., Garnier C. (2019). Extracellular matrix contribution to skin wound re-epithelialization. Matrix Biol. J. Int. Soc. Matrix Biol..

[28] Smola H., Thiekotter G., Fusenig N. (1993). Mutual induction of growth factor gene expression in by epidermal-dermal cell interaction. J. Cell Biol..

[29] Piipponen M., Li D., Landén N.X. (2020). The Immune Functions of Keratinocytes in Skin Wound Healing. Int. J. Mol. Sci..

[30] Azari Z., Nazarnezhad S., Webster T.J., Hoseini S.J., Brouki Milan P., Baino F., Kargozar S. (2022). Stem cell-mediated angiogenesis in skin tissue engineering and wound healing. Wound Rep. Regen. Off. Publ. Wound Heal. Soc. Eur. Tissue Rep. Soc..

[31] Veith A.P., Henderson K., Spencer A., Sligar A.D., Baker A.B. (2019). Therapeutic strategies for enhancing angiogenesis in wound healing. Adv. Drug Deliv. Rev..

[32] Talbott H.E., Mascharak S., Griffin M., Wan D.C., Longaker M.T. (2022). Wound healing, fibroblast heterogeneity, and fibrosis. Cell Stem Cell.

[33] Barker T.H. (2011). The role of ECM proteins and protein fragments in guiding cell behavior in regenerative medicine. Biomaterials.

[34] Eckes B., Nischt R., Krieg T. (2010). Cell-matrix interactions in dermal repair and scarring. Fibrogenes. Tissue Rep..

[35] El Ayadi A., Jay J.W., Prasai A. (2020). Current Approaches Targeting the Wound Healing Phases to Attenuate Fibrosis and Scarring. Int. J. Mol. Sci..

[36] Sharma S., Rai V.K., Narang R.K., Markandeywar T.S. (2022). Collagen-based formulations for wound healing: A literature review. Life Sci..

[37] Kandhwal M., Behl T., Singh S., Sharma N., Arora S., Bhatia S., Al-Harrasi A., Sachdeva M., Bungau S. (2022). Role of matrix metalloproteinase in wound healing. Am. J. Transl. Res..

[38] Margolis D.J., Hoffstad O., Nafash J., Leonard C.E., Freeman C.P., Hennessy S., Wiebe D.J. (2011). Location, location, location: Geographic clustering of lower-extremity amputation among Medicare beneficiaries with diabetes. Diabetes Care.

[39] Stiehl J.B. (2021). Early wound bed preparation: Irrigation and debridement. J. Wound Care.

[40] Maemoto R., Noda H., Ichida K., Miyakura Y., Kakizawa N., Machida E., Aizawa H., Kato T., Iseki M., Fukui T. (2022). Aqueous Povidone-Iodine Versus Normal Saline For Intraoperative Wound Irrigation On The Incidence Of Surgical Site Infection In Clean-Contaminated Wounds After Gastroenterological Surgery: A Single Institute, Prospective, Blinded-Endpoint, Randomized Controlled Trial. Ann. Surg..

[41] Rai S., Gupta T.P., Shaki O., Kale A. (2021). Hydrogen Peroxide: Its Use in an Extensive Acute Wound to Promote Wound Granulation and Infection Control—Is it Better Than Normal Saline?. Int. J. Low. Extrem. Wounds.

[42] Powers J.G., Higham C., Broussard K., Phillips T.J. (2016). Wound healing and treating wounds: Chronic wound care and management. J. Am. Acad. Dermatol..

[43] Obagi Z., Damiani G., Grada A., Falanga V. (2019). Principles of Wound Dressings: A Review. Surg. Technol. Int..

[44] Braza M.E., Fahrenkopf M.P. (2022). Split-Thickness Skin Grafts. StatPearls.

[45] Holl J., Kowalewski C., Zimek Z., Fiedor P., Kaminski A., Oldak T., Moniuszko M., Eljaszewicz A. (2021). Chronic Diabetic Wounds and Their Treatment with Skin Substitutes. Cells.

[46] Hekmatpou D., Mehrabi F., Rahzani K., Aminiyan A. (2019). The Effect of Aloe Vera Clinical Trials on Prevention and Healing of Skin Wound: A Systematic Review. Iran. J. Med. Sci..

[47] Kumar R., Singh A.K., Gupta A., Bishayee A., Pandey A.K. (2019). Therapeutic potential of Aloe vera-A miracle gift of nature. Phytomed. Int. J. Phytother. Phytopharmacol..

[48] Tam J.C., Ko C.H., Zhang C., Wang H., Lau C.P., Chan W.Y., Leung P.C., Fung K.P., Zhang J.F., Lau C.B. (2014). Comprehensive proteomic analysis of a Chinese 2-herb formula (Astragali Radix and Rehmanniae Radix) on mature endothelial cells. Proteomics.

[49] Tam J.C., Ko C.H., Lau K.M., To M.H., Kwok H.F., Siu W.S., Lau C.P., Chan W.Y., Leung P.C., Fung K.P. (2015). Enumeration and functional investigation of endothelial progenitor cells in neovascularization of diabetic foot ulcer rats with a Chinese 2-herb formula. J. Diabetes.

[50] Ko C.H., Yi S., Ozaki R., Cochrane H., Chung H., Lau W., Koon C.M., Hoi S.W., Lo W., Cheng K.F. (2014). Healing effect of a two-herb recipe (NF3) on foot ulcers in Chinese patients with diabetes: A randomized double-blind placebo-controlled study. J. Diabetes.

[51] Naji A., Eitoku M., Favier B., Deschaseaux F., Rouas-Freiss N., Suganuma N. (2019). Biological functions of mesenchymal stem cells and clinical implications. Cell. Mol. Life Sci..

[52] Kumar P., Kandoi S., Misra R., Vijayalakshmi S., Rajagopal K., Verma R.S. (2019). The mesenchymal stem cell secretome: A new paradigm towards cell-free therapeutic mode in regenerative medicine. Cytokine Growth Factor Rev..

[53] Dominici M., Le Blanc K., Mueller I., Slaper-Cortenbach I., Marini F., Krause D., Deans R., Keating A., Prockop D.j., Horwitz E. (2006). Minimal criteria for defining multipotent mesenchymal stromal cells. The International Society for Cellular Therapy position statement. Cytotherapy.

[54] Mishra V.K., Shih H.H., Parveen F., Lenzen D., Ito E., Chan T.F., Ke L.Y. (2020). Identifying the Therapeutic Significance of Mesenchymal Stem Cells. Cells.

[55] Xunian Z., Kalluri R. (2020). Biology and therapeutic potential of mesenchymal stem cell-derived exosomes. Cancer Sci..

[56] Mazini L., Rochette L., Admou B., Amal S., Malka G. (2020). Hopes and Limits of Adipose-Derived Stem Cells (ADSCs) and Mesenchymal Stem Cells (MSCs) in Wound Healing. Int. J. Mol. Sci..

[57] Gnecchi M., He H., Noiseux N., Liang O.D., Zhang L., Morello F., Mu H., Melo L.G., Pratt R.E., Ingwall J.S. (2006). Evidence supporting paracrine hypothesis for Akt-modified mesenchymal stem cell-mediated cardiac protection and functional improvement. FASEB J..

[58] Ma H., Lam P.K., Siu W.S., Tong C.S.W., Lo K.K.Y., Koon C.M., Wu X.X., Li X., Cheng W., Shum W.T. (2021). Adipose Tissue-Derived Mesenchymal Stem Cells (ADMSCs) and ADMSC-Derived Secretome Expedited Wound Healing in a Rodent Model—A Preliminary Study. Clin. Cosmet. Investig. Dermatol..

[59] Stojanović S., Najman S. (2019). The Effect of Conditioned Media of Stem Cells Derived from Lipoma and Adipose Tissue on Macrophages’ Response and Wound Healing in Indirect Co-culture System In Vitro. Int. J. Mol. Sci..

[60] Ahangar P., Mills S.J., Smith L.E., Strudwick X.L., Ting A.E., Vaes B., Cowin A.J. (2020). Human multipotent adult progenitor cell-conditioned medium improves wound healing through modulating inflammation and angiogenesis in mice. Stem Cell Res. Ther..

[61] Kim J.H., Green D.S., Ju Y.M., Harrison M., Vaughan J.W., Atala A., Lee S.J., Jackson J.D., Nykiforuk C., Yoo J.J. (2022). Identification and characterization of stem cell secretome-based recombinant proteins for wound healing applications. Front. Bioeng. Biotechnol..

[62] Ma H., Siu W.S., Koon C.M., Wu X.X., Li X., Cheng W., Shum W.T., Lau C.B., Wong C.K., Leung P.C. (2023). The Application of Adipose Tissue-Derived Mesenchymal Stem Cells (ADMSCs) and a Twin-Herb Formula to the Rodent Wound Healing Model: Use Alone or Together?. Int. J. Mol. Sci..

[63] Ahangar P., Mills S.J., Cowin A.J. (2020). Mesenchymal Stem Cell Secretome as an Emerging Cell-Free Alternative for Improving Wound Repair. Int. J. Mol. Sci..

[64] Ferreira J.R., Teixeira G.Q., Santos S.G., Barbosa M.A., Almeida-Porada G., Gonçalves R.M. (2018). Mesenchymal Stromal Cell Secretome: Influencing Therapeutic Potential by Cellular Pre-conditioning. Front. Immunol..

[65] Zhang J., Zhou R., Xiang C., Jia Q., Wu H., Yang H. (2020). Huangbai Liniment Accelerated Wound Healing by Activating Nrf2 Signaling in Diabetes. Oxid. Med. Cell. Longev..

[66] Kehl D., Generali M., Mallone A., Heller M., Uldry A.C., Cheng P., Gantenbein B., Hoerstrup S.P., Weber B. (2019). Proteomic analysis of human mesenchymal stromal cell secretomes: A systematic comparison of the angiogenic potential. NPJ Regen. Med..

[67] Maacha S., Sidahmed H., Jacob S., Gentilcore G., Calzone R., Grivel J.C., Cugno C. (2020). Paracrine Mechanisms of Mesenchymal Stromal Cells in Angiogenesis. Stem Cells Int..

[68] Hsiao S.T., Asgari A., Lokmic Z., Sinclair R., Dusting G.J., Lim S.Y., Dilley R.J. (2012). Comparative analysis of paracrine factor expression in human adult mesenchymal stem cells derived from bone marrow, adipose, and dermal tissue. Stem Cells Dev..

[69] Guillamat-Prats R. (2021). The Role of MSC in Wound Healing, Scarring and Regeneration. Cells.

[70] Saheli M., Bayat M., Ganji R., Hendudari F., Kheirjou R., Pakzad M., Najar B., Piryaei A. (2020). Human mesenchymal stem cells-conditioned medium improves diabetic wound healing mainly through modulating fibroblast behaviors. Arch. Dermatol. Res..

[71] Campanella C., Caruso Bavisotto C., Logozzi M., Marino Gammazza A., Mizzoni D., Cappello F., Fais S. (2019). On the Choice of the Extracellular Vesicles for Therapeutic Purposes. Int. J. Mol. Sci..

[72] Pan B.T., Johnstone R.M. (1983). Fate of the transferrin receptor during maturation of sheep reticulocytes in vitro: Selective externalization of the receptor. Cell.

[73] Toh W.S., Lai R.C., Zhang B., Lim S.K. (2018). MSC exosome works through a protein-based mechanism of action. Biochem. Soc. Trans..

[74] Théry C., Witwer K.W., Aikawa E., Alcaraz M.J., Anderson J.D., Andriantsitohaina R., Antoniou A., Arab T., Archer F., Atkin-Smith G.K. (2018). Minimal information for studies of extracellular vesicles 2018 (MISEV2018): A position statement of the International Society for Extracellular Vesicles and update of the MISEV2014 guidelines. J. Extracell. Vesicles.

[75] Borges F.T., Convento M.B., Schor N. (2018). Bone marrow-derived mesenchymal stromal cell: What next?. Stem Cells Cloning.

[76] Blazquez R., Sanchez-Margallo F.M., de la Rosa O., Dalemans W., Alvarez V., Tarazona R., Casado J.G. (2014). Immunomodulatory Potential of Human Adipose Mesenchymal Stem Cells Derived Exosomes on in vitro Stimulated T Cells. Front. Immunol..

[77] Dalirfardouei R., Jamialahmadi K., Jafarian A.H., Mahdipour E. (2019). Promising effects of exosomes isolated from menstrual blood-derived mesenchymal stem cell on wound-healing process in diabetic mouse model. J. Tissue Eng. Regen. Med..

[78] Vu N.B., Nguyen H.T., Palumbo R., Pellicano R., Fagoonee S., Pham P.V. (2021). Stem cell-derived exosomes for wound healing: Current status and promising directions. Minerva Med..

[79] He X., Dong Z., Cao Y., Wang H., Liu S., Liao L., Jin Y., Yuan L., Li B. (2019). MSC-Derived Exosome Promotes M2 Polarization and Enhances Cutaneous Wound Healing. Stem Cells Int..

[80] Ti D., Hao H., Tong C., Liu J., Dong L., Zheng J., Zhao Y., Liu H., Fu X., Han W. (2015). LPS-preconditioned mesenchymal stromal cells modify macrophage polarization for resolution of chronic inflammation via exosome-shuttled let-7b. J. Transl. Med..

[81] Li X., Liu L., Yang J., Yu Y., Chai J., Wang L., Ma L., Yin H. (2016). Exosome Derived From Human Umbilical Cord Mesenchymal Stem Cell Mediates MiR-181c Attenuating Burn-induced Excessive Inflammation. EBioMedicine.

[82] Ha D.H., Kim H.K., Lee J., Kwon H.H., Park G.H., Yang S.H., Jung J.Y., Choi H., Lee J.H., Sung S. (2020). Mesenchymal Stem/Stromal Cell-Derived Exosomes for Immunomodulatory Therapeutics and Skin Regeneration. Cells.

[83] Shabbir A., Cox A., Rodriguez-Menocal L., Salgado M., Van Badiavas E. (2015). Mesenchymal Stem Cell Exosomes Induce Proliferation and Migration of Normal and Chronic Wound Fibroblasts, and Enhance Angiogenesis In Vitro. Stem Cells Dev..

[84] Zhang B., Wang M., Gong A., Zhang X., Wu X., Zhu Y., Shi H., Wu L., Zhu W., Qian H. (2015). HucMSC-Exosome Mediated-Wnt4 Signaling Is Required for Cutaneous Wound Healing. Stem Cells.

[85] Yang C., Luo L., Bai X., Shen K., Liu K., Wang J., Hu D. (2020). Highly-expressed micoRNA-21 in adipose derived stem cell exosomes can enhance the migration and proliferation of the HaCaT cells by increasing the MMP-9 expression through the PI3K/AKT pathway. Arch. Biochem. Biophys..

[86] Zheng T., Shao W., Tian J. (2021). Exosomes derived from ADSCs containing miR-378 promotes wound healing by targeting caspase-3. J. Biochem. Mol. Toxicol..

[87] Cao G., Chen B., Zhang X., Chen H. (2020). Human Adipose-Derived Mesenchymal Stem Cells-Derived Exosomal microRNA-19b Promotes the Healing of Skin Wounds Through Modulation of the CCL1/TGF-β Signaling Axis. Clin. Cosmet. Investig. Dermatol..

[88] Pi L., Yang L., Fang B.R., Meng X.X., Qian L. (2022). LncRNA MALAT1 from human adipose-derived stem cell exosomes accelerates wound healing via miR-378a/FGF2 axis. Regen. Med..

[89] Ren S., Chen J., Duscher D., Liu Y., Guo G., Kang Y., Xiong H., Zhan P., Wang Y., Wang C. (2019). Microvesicles from human adipose stem cells promote wound healing by optimizing cellular functions via AKT and ERK signaling pathways. Stem Cell Res. Ther..

[90] Yang K., Li D., Wang M., Xu Z., Chen X., Liu Q., Sun W., Li J., Gong Y., Liu D. (2019). Exposure to blue light stimulates the proangiogenic capability of exosomes derived from human umbilical cord mesenchymal stem cells. Stem Cell Res. Ther..

[91] Ding J., Wang X., Chen B., Zhang J., Xu J. (2019). Exosomes Derived from Human Bone Marrow Mesenchymal Stem Cells Stimulated by Deferoxamine Accelerate Cutaneous Wound Healing by Promoting Angiogenesis. Biomed. Res. Int..

[92] Lintel H., Abbas D.B., Lavin C.V., Griffin M., Guo J.L., Guardino N., Churukian A., Gurtner G.C., Momeni A., Longaker M.T. (2022). Transdermal deferoxamine administration improves excisional wound healing in chronically irradiated murine skin. J. Transl. Med..

[93] Zhang Y., Pan Y., Liu Y., Li X., Tang L., Duan M., Li J., Zhang G. (2021). Exosomes derived from human umbilical cord blood mesenchymal stem cells stimulate regenerative wound healing via transforming growth factor-β receptor inhibition. Stem Cell Res. Ther..

[94] Rozier P., Maumus M., Maria A.T.J., Toupet K., Lai-Kee-Him J., Jorgensen C., Guilpain P., Noël D. (2021). Mesenchymal stromal cells-derived extracellular vesicles alleviate systemic sclerosis via miR-29a-3p. J. Autoimmun..

[95] Oualla-Bachiri W., Fernández-González A., Quiñones-Vico M.I., Arias-Santiago S. (2020). From Grafts to Human Bioengineered Vascularized Skin Substitutes. Int. J. Mol. Sci..

[96] Wahlberg B., Ghuman H., Liu J.R., Modo M. (2018). Ex vivo biomechanical characterization of syringe-needle ejections for intracerebral cell delivery. Sci. Rep..

[97] Abbasi-Malati Z., Roushandeh A.M., Kuwahara Y., Roudkenar M.H. (2018). Mesenchymal Stem Cells on Horizon: A New Arsenal of Therapeutic Agents. Stem Cell Rev. Rep..

[98] Torizal F.G., Fiano F., Khumaira A. (2022). Production of mesenchymal stem cell derived-secretome as cell-free regenerative therapy and immunomodulation: A biomanufacturing perspective. Biocell.

[99] Teixeira F.G., Salgado A.J. (2020). Mesenchymal stem cells secretome: Current trends and future challenges. Neural Regen. Res..

[100] Sagaradze G., Grigorieva O., Nimiritsky P., Basalova N., Kalinina N., Akopyan Z., Efimenko A. (2019). Conditioned Medium from Human Mesenchymal Stromal Cells: Towards the Clinical Translation. Int. J. Mol. Sci..

[101] Park S., Lee D.R., Nam J.S., Ahn C.W., Kim H. (2018). Fetal bovine serum-free cryopreservation methods for clinical banking of human adipose-derived stem cells. Cryobiology.

